# Comparing the average cost of outpatient care of public and for-profit private providers in India

**DOI:** 10.1186/s12913-021-06777-7

**Published:** 2021-08-19

**Authors:** Samir Garg, Narayan Tripathi, Alok Ranjan, Kirtti Kumar Bebarta

**Affiliations:** 1 State Health Resource Centre, Raipur, Chhattisgarh India; 2 Indian Institute of Technology, Jodhpur, India

## Abstract

**Introduction:**

Understanding the cost of care associated with different kinds of healthcare providers is necessary for informing the policy debates in mixed health-systems like India’s. Existing studies reporting Out of Pocket Expenditure (OOPE) per episode of outpatient care in public and private providers in India do not provide a fair comparison because they have not taken into account the government subsidies received by public facilities. Public and private health insurance in India do not cover outpatient care and for-profit providers have to meet all their costs out of the payments they take from patients.

**Methods:**

The average direct cost per acute episode of outpatient care was compared for public providers, for-profit formal providers and informal private providers in Chhattisgarh state of India. For public facilities, government subsidies for various inputs were taken into account. Resources used were apportioned using Activity Based Costing. Land provided free to public facilities was counted at market prices. The study used two datasets: a) household survey on outpatient utilisation and OOPE b) facility survey of public providers to find the input costs borne by government per outpatient-episode.

**Results:**

The average cost per episode of outpatient care was Indian Rupees (INR) 400 for public providers, INR 586 for informal private providers and INR 2643 for formal for-profit providers and they managed 39.3, 37.9 and 22.9% of episodes respectively. The average cost for government and households put together was greater for using formal for-profit providers than the public providers. The disease profile of care handled by different types of providers was similar. Volume of patients and human-resources were key cost drivers in public facilities. Close to community providers involved less cost than others.

**Conclusions and recommendations:**

The findings have implications for the desired mix of public and private providers in India’s health-system. Poor regulation of for-profit providers was an important structural cost driver. Purchasing outpatient care from private providers may not reduce average cost. Policies to strengthen public provisioning of curative primary care close to communities can help in reducing cost.

**Supplementary Information:**

The online version contains supplementary material available at 10.1186/s12913-021-06777-7.

## Introduction

Understanding the cost of care associated with different kinds of healthcare providers is necessary for informing the policy debates in mixed health systems [1–3]. There is a paucity of such information in most Low- and Medium Income Countries (LMICs) including India [1, 2]. India has a mixed health system with a sizeable presence of private sector. The formal and informal private providers together accounted for 69.8% of India’s outpatient care, the most common form of healthcare utilisation [4].

A couple of studies in India have compared the costs for public and private providers. These studies have examined the unit costs of healthcare delivery for the public as well as private hospitals [5, 6]. These studies report the cost of delivering healthcare in terms of the average cost of inputs consumed per episode of care. They do not report the total cost of per episode of care. The Out of Pocket Expenditure (OOPE) incurred by the patients has not been considered in these studies [5, 6].

Another set of studies in India have focused on comparing the OOPE per episode of care in public and private facilities. These studies show that the OOPE incurred per episode in private sector was substantially higher than in public sector [7, 8]. However, such comparisons may not portray a fair picture because they do not take into account the government subsidies received by public facilities. Services from for-profit private providers are bound to be more expensive for the patient because private providers do not get the government subsidies that public facilities enjoy. A fair comparison of cost of care thus requires that apart from OOPE, the cost borne by government is also taken into account [9].

In India, government provides budget based financing to public facilities, covering a large share of their input costs [10]. India’s publicly funded health insurance schemes do not cover outpatient care [11]. Private health insurance in India is also limited to in-patient care [12]. For-profit private facilities providing outpatient care in India thus have to cover all their input costs out of the payments they take from patients.

This study is aimed at carrying out a fair comparison of average cost of acute outpatient care in different types of outpatient healthcare providers for a state in India. It aims to take into account all the direct costs borne by patients and government. The study also seeks to provide a comparison with informal private providers who constitute a significant part of outpatient utilisation in India but have not received much attention in recent studies of healthcare costs in India.

## Methods

### Study area

This study was conducted in Chhattisgarh, one of the poorest states in India [13]. The state had a population of around 28 million in 2019, with 77% of it living in rural areas [11].

### Study design and key concepts

This was a descriptive study. The study relied on two datasets:

- A survey on utilisation of outpatient care by households and individuals including the type of illness, type of providers utilised and OOPE: A large household survey on morbidity and utilisation was carried out in Chhattisgarh in November-December 2019. This survey was carried out by the State Health Resource Centre, a technical agency working for the Department of Health, Government of Chhattisgarh. The survey had a representative sample of state’s population, covering 1500 households with 8286 individuals. It was a two-stage randomized sample. It covered all the five geographical divisions of the state. The survey included data about self-reported acute ailments in last fifteen days, the type of care sought for them and OOPE.
- A survey of government health facilities to find their input costs: A survey of various kinds of government facilities was carried out in early 2020. For this facility survey, a representative sample of facilities was taken out of the government facilities from the areas as covered in the above mentioned household survey.

The health system of Chhattisgarh like most states of India is organized in different tiers - a Sub Health Centre (SHC) at 3000 to 5000 population, a Primary Health Centre (PHC) at 20,000 to 30,000 population, a Community health Centre at 80,000 to 120,000 population and a District Hospital (DH) at around a million population. DH and CHC provide secondary and primary care whereas PHCs and SHCs focus exclusively on primary care [14]. All these facilities handle three kinds of functions broadly: a) providing outpatient care b) providing inpatient care c) non-clinical public health functions. In addition, there are Community Health Workers (CHWs) looking at an average population of around 350, who are paid by government. Along with their role in preventive health, the CHWs are also trained to provide curative care at community level for specific ailments [15].

From each of the five districts covered in household survey; one DH, three CHCs, four PHCs, five SHCs and ten CHWs were selected randomly for this facility-survey.

Ethics approval for the study was obtained from the Institutional Ethics Committee of State Health Resource Centre, Chhattisgarh.

### Type of providers

The providers from whom healthcare was received by individuals were classified into three main categories:

- ***Public providers*:** It included all government owned facilities as well as the CHWs paid by government.
- ***Private For-Profit Formal Providers:*** This included the qualified for-profit providers with formal registration.
- ***Informal Private Providers:*** This group included a variety of informal providers without medical education. They operated outside the law because they did not have the qualifications to get registered under the state’s law for clinical establishments [16].

The disease profile of outpatient episodes handled by the three types of providers was compared using ANOVA.

### Out of pocket expenditure

Out of pocket Expenditure (OOPE) was taken as the direct expenses incurred by the individual/family during the episode on a) Medical expenses: paying the provider fees, buying drugs or tests; b) Non-medical expenses: food and transport of patient and attendants. Any reimbursements or incentive payments received by the patient were subtracted to calculate the OOPE [17]. Data on OOPE was obtained from the household survey. In episodes where the primary utilisation of outpatient-care i.e. physician consultation was with a public provider but drugs or tests were bought by patient from outside, the OOPE incurred was counted under utilisation of public providers.

Cross-tabulations were used for descriptive comparison of OOPE for different kinds of providers. Linear regression was performed to find the determinants of size of OOPE.

### Government expenditure for inputs in public facilities for providing outpatient care

The purpose of the study was to find out total cost of all resources used per episode of outpatient care. For this purpose, both Activity Based Costing (ABC) as well as Time-Driven Activity Based Costing (TD-ABC) was suitable. ABC was applied to apportion the different resources to outpatient care.

Under ABC, government expenditure was computed using the facility survey data on basic inputs including recurring costs of human resources (HR), drugs, consumables etc. Cost of land was taken at current open market prices. Costs of capital items like land, building and equipments were annualized by using ‘discount rates’ and expected life-span [18]. Land was discounted at 7% per annum, considering that the market cost of borrowing capital in India is around 7%. The useful life of buildings was taken as 25 years and building cost was discounted at 11% [18–20]. The useful life of equipments was taken as 5 years and their cost was discounted at 27%.

Detailed cost data was collected for twenty items. They were classified into six types of main resources. The total of input costs was apportioned to outpatient care based on the proportion of working time spent by the facility’s staff on outpatient care and related services. This method of apportioning the total cost of a hospital to a specific function (e.g. outpatient care) has been used by many studies in India [18–20]. In the public facilities included in this study, most of the staff and infrastructure were deployed for multiple functions like outpatient care, inpatient care and non-clinical activities. Therefore, counting the costs separately for outpatient care alone was not possible in this context. According to secondary literature, human resources constitute the biggest component of cost in health facilities in India [18]. Therefore, the above strategy of apportioning based on staff time was used. The per-episode average was computed by dividing the total government cost on outpatient care by the annual number of episodes for each type of public facility.

A detailed note on the steps used for costing is given in Additional file S1. Sensitivity analysis was carried out to see the impact on average cost by changing the key assumptions – by changing the discount rates used for annualizing capital costs, proportion of cost of different inputs apportioned to outpatient care and volume of patients seen by the facility (Additional file S3). For this analysis, the cost-components were changed by 30% on either side to understand their impact.

For robustness, costing was also carried out by using the Time-Driven Activity Based Costing (TD-ABC) method. This method has been devised to overcome the limitations of ABC [21–23]. It has been found useful for finding cost per patient in general healthcare situations like the current study [24, 25]. A process map was prepared for outpatient care (Additional file S4). Based on the processes involved in outpatient care, time equations were developed to find the minutes of staff time required per outpatient episode. Cost per minute of practical staff capacity was used to calculate the total cost per episode of outpatient-care.

### Total average direct cost of care

As defined in literature, total direct cost was taken as the sum of costs borne by the government, community and patients [26–30]. The approach was to find the total cost by adding the costs incurred by different actors while ensuring that no cost gets counted twice [28–31].

Total Average Direct Cost of Care in public facilities was taken as the sum of: a) Average Government expenditure for inputs in public facilities and b) Mean OOPE incurred by patient/family.

For private providers, OOPE incurred by patients was taken as the Total Direct Cost of Care. For-profit private providers meet their input costs out of the payments taken from patients. The expenditure by private facilities on inputs used was thus not added to OOPE because it would amount to double counting of these costs.

The study did not include the indirect or intangible costs i.e. the loss of productivity or income due to episodes of illness. There were no insurance costs or reimbursements involved as public and private health insurance in India did not include outpatient care.

## Results

The key demographic characteristics of the sample of household survey are given in Additional file S2.

### Type of providers used

The proportion of outpatient episodes handled by different providers is given in Table 1.

**Table 1:** Outpatient episodes handled by different types of providers

Type of Provider	Share in utilisation of outpatient care (%) (***N*** = 809 episodes)
*Public Providers:*	
CHWs	17.8
SHC	4.9
PHC	9.6
CHC	5.3
DH	1.7
**All Public Providers**	**39.3**
*Formal For-Profit Providers*:	
Private Hospital	8.5
Private Clinic	14.4
**All Formal For-Profit Providers**	**22.9**
*Informal Private Providers*:	
Private Pharmacy	3.4
Informal Allopathic Providers	32.8
Traditional healers	1.7
**All Informal Private Providers**	**37.9**

Among the three types of providers, the public providers had the biggest share, followed closely by the informal private providers (Table 1). The formal for-profit private sector had the smallest share in utilisation. Among public providers, CHWs had a bigger share than others. Among the formal for-profit providers, private clinics accounted for greater share of utilisation than the private hospitals.

The disease-wise break-up of episodes of acute outpatient care handled by public and private providers is given in Table 2. Typhoid was the only disease that formed a greater share of formal private sector utilisation in comparison to public providers (*p* < 0.05). Rest all the diseases had similar proportions in the disease profile of different providers.

**Table 2:** Proportion of Different Ailments in the Outpatient care Episodes managed by different Types of Providers

Disease	Public Providers	Formal For-Profit Providers	Informal Private Providers	***p*** value
	(***N*** = 318)	(***N*** = 185)	(***N*** = 306)	
Cold and cough	57.1	64.8	66.4	0.086
Malaria	15.5	11.5	12.5	0.109
Diarrhea	5.6	3.1	2.3	0.074
Body Pain	5.0	3.5	3.9	0.298
Skin Infection	3.1	2.9	3.0	0.715
Typhoid	1.9	4.1	2.3	0.002*
Knee and Joint Pain	1.6	0.6	0.3	0.313
Weakness	1.6	0.8	1.0	0.575
Injury	1.6	0.8	1.0	0.861
Respiratory Infection	0.9	0.4	1.0	0.639
Stomach ache	0.9	1.2	0.1	0.284
Others	5.3	6.2	6.3	0.629
Total	100	100	100	

*p < 0.05

In addition to the episodes reported in Table 2, there were 12 episodes of outpatient care that took place in charitable institutions i.e. the not-for-profit facilities. They were not included in the analysis.

### Average government expenditure on inputs per episode of outpatient care in public facilities

Table 3 provides the Average Government Expenditure per episode of outpatient care in different kinds of public facilities. Table 3 shows that the government subsidy per episode was highest for CHCs followed by PHCs. The tables on detailed cost structure for each type of public facility are given in Additional file S1.

**Table 3:** Cost per episode of outpatient care of public and for-profit private providers

Type of Provider	Cost per episode of Outpatient Care		
	Average Government expenditure (INR)	Average OOPE (INR)	Total Average Direct Cost (INR)
	(A)	(B)	(A + B)
*Public Providers:*			
CHWs	54	41	95
SHC	184	55	239
PHC	358	276	634
CHC	373	504	877
DH	305	993	1298
**All Public Providers**	**198**	**202**	**400**
*Formal For-Profit Providers*:			
Private Hospital	0	3323	3323
Private Clinic	0	2243	2243
**All Formal For-Profit Providers**	**0**	**2643**	**2643**
*Informal Private Providers*:			
Private Pharmacy	0	497	497
Informal Allopathic Providers	0	598	598
Traditional healers	0	539	539
**All Informal Private Providers**	**0**	**586**	**586**

#### Average OOPE

Table 3 provides the average OOPE per episode for utilisation in different types of public and private facilities. A disease-category wise comparison of OOPE for different providers is given in Additional file S5.

Linear regression performed for determinants of size of OOPE showed that an episode of outpatient care with formal private providers was significantly likely to involve OOPE of INR 2034 greater than in public facilities (Additional file S6). However, the regression showed that OOPE for the informal private providers was not significantly different from the public providers.

Sensitivity analysis showed that the average cost in public facilities was most sensitive to volume of patients seen, followed by the human resources and infrastructure cost (Additional file S3).

Using the TD-ABC method, the average cost per patient in public facilities was INR 112, which was 57% of the cost INR 198 found through ABC. This ratio was lowest for PHCs (40%) and highest for DHs (90%).

Cost Drivers: According to ABC, human resources and infrastructure were the main cost drivers in public facilities. Human resources contributed to 53% of the average cost followed by infrastructure at 24% share. The share of other main types of resources was medicines (11%) and diagnostics (3%). A break-up of costs for different types of public facilities according to type of resource used is given in Additional file S1.

#### Average direct cost of care

Table 3 provides the Average Direct Cost of Care per episode of outpatient care in different types of facilities.

In terms of the total average cost for an outpatient-episode in public facilities, DH was costliest followed by CHCs and then PHCs (Table 3). Among the public providers, CHWs had the lowest total average cost followed by SHCs. Informal private providers had lower costs than the formal private providers. Within formal for-profit providers, private clinics had lower costs than the private hospitals.

## Discussion

The current study found that the total average cost per episode of outpatient care for public providers was INR 400. This included average OOPE of INR 202 per episode. A national survey in 2017-18 had reported a similar amount of OOPE in public facilities [4, 7].

The public facilities spent INR 198 per episode of outpatient care on inputs like human resources, supplies, annual cost of infrastructure etc. This cost on inputs in public facilities was met through budgetary support from government. Earlier studies have calculated these costs using similar methods. A recent study of three states of India has reported the input cost of outpatient care per episode as INR 183 for DH and INR 134 for CHC [18]. The current study found the above cost was higher in Chhattisgarh - INR 305 for DH and INR 373 for CHC. Earlier studies had found that outpatient care constituted around 20% of the total hospital cost whereas the share apportioned for outpatient care in the current study was greater, at 41.4% for DH and 36.5% for CHC [5].

Recently, da Viega et al. have applied the strategic cost management perspective in their study of cost of care in Brazilian public and private systems. They have analysed the cost-drivers at two levels - executional and structural. While the executional cost-drivers are relevant for deciding short term tactics to improve process efficiency, the structural cost-drivers relate to long-term and strategic issues in the larger system or value-chain [23]. In the current study, the patient volume in relation to human resources was an executional cost-driver in public facilities. Application of TD-ABC also showed the gap in capacity utilisation, especially in PHCs.

The current study found that among the public providers, primary care providers delivering care close to community involved less cost than the secondary care providers. Earlier studies in India have also reported a similar pattern [32, 33]. This suggests that organizing primary care close to where people live can help in bringing down the costs for government and patients.

The current study found that among the for-profit providers, the informal providers were less costly than formal providers. Earlier studies in India have not examined the cost associated with care from informal private providers. This was a serious gap in existing literature considering the large share informal providers command in outpatient care in India. There can be valid concerns regarding the quality of care provided by informal providers, given their lack of medical training [34]. This suggests the need for more effective regulation of such medical practice.

One of the reasons for the popularity of informal providers in India has been their accessibility [35]. The quality of care can improve if conditions are created for patients to shift from informal private providers to public providers. In this regard, India’s recent policy of Comprehensive Primary Health Care (CPHC) bears significance. As a part of this policy, Indian government aims to start public clinics known as Health and Wellness Centres at 5000 population [36].

The main purpose of the current study was to attempt a fair comparison of average cost of outpatient care in public and for-profit private providers. OOPE for using private providers is expected to be greater because they need to earn a profit to stay afloat whereas government services can even be free. Was the comparison fair then? In order to enable a fair comparison, the total cost in this study took into account the government subsidies to public facilities. The study tried to find out what it would cost to government and households put together if an episode of outpatient care were to take place in a public facility, as compared to a private one. It found that such average cost was six times lower in the public sector than the formal for-profit sector.

A potential explanation for higher costs of formal private facilities could be that they perhaps invest more than the public facilities in inputs like infrastructure and human resources. However, there is no conclusive evidence of for-private sector providing better quality of care than the public sector globally [37–39].

Another factor contributing to high OOPE in for-profit sector could be related to its tendency to push unnecessary and costly drugs and tests. Several studies from different parts of the world have suggested this possibility [40–48]. Many studies from India also report similar findings [8, 49–57].

Many studies of private providers have reported that over-charging of patients was a common practice in India [8, 56–61]. The practice of overcharging or price gouging has been reported from some High Income Countries (HICs) too [62]. Some reviews from HICs have concluded that the charges taken by for-profit providers tend to be much higher than their cost of production [63]. Such findings can have implications for the allocative efficiency in health systems [63]. The current study suggests that this might be the case in India too.

India does not have effective regulation of cost or quality in private healthcare sector [8, 50]. The poor regulation of private providers emerged as the key ‘structural’ cost-driver in the current study. Lack of regulation can result in providers charging excessively. Regulation has been identified as one of the potential cost-drivers at health-systems level [64]. Some have taken the analysis further and attributed the poor regulation in mixed health-systems in LMICs to deepening of power-asymmetries under market based healthcare [40, 56].

A common policy prescription for LMICs including India has been to purchase care from private sector using mechanisms like publicly funded insurance. India has implemented such policies for purchasing inpatient care, but with little success in reducing OOPE [11, 65–69]. Yet, many have advocated that publicly funded insurance should be extended to primary care [68, 69]. The current study suggests extending such arrangements to outpatient care can result in increasing the average cost of care substantially.

Studies in India and other LMICs have found that public sector tends to under-provide certain services because of being under-funded [23, 57]. In the current study, government spent a very small amount on providing diagnostics. How would the costs change if public providers started providing the required diagnostic tests adequately? The sensitivity analysis showed that a substantial rise in government spending on diagnostics will increase the overall cost marginally. This shows the need to increase public funding for providing essential diagnostics in government facilities. This may also help them in attracting more patients.

Chhattisgarh, like many states in India has implemented a policy to provide essential drugs free of cost in its public facilities [70]. Expenditure on drugs has been known to be a key cause of OOPE in India. Providing drugs free thus could have helped in bringing down the OOPE considerably. For keeping the government spending on drugs within its means, Chhattisgarh had set up a mechanism for bulk purchase of drugs following the successful examples of other states in India like Tamil Nadu and Rajasthan [71]. Similar arrangements have shown benefits of cost saving in other South Asian countries like Thailand [72]. The current study suggests that strengthening such policies can help in reducing the average cost of care. Another aspect related to cost of medicines used is of whether branded or generic versions were used. The branded versions are usually several times more expensive than the generic version. Private providers mostly prescribe branded versions. The state covered in the current study does not allow the public providers to prescribe branded medicines [70].

The current study adds to the sparse literature available on cost comparisons between different kinds of healthcare providers in India and other low-income countries. It also fills a gap in literature regarding cost of care for informal private providers. Further, by providing a valid comparison between different providers the current study makes a contribution to policy debates on public-private provider mix in India.

The study makes important contribution in methodological aspects. This is the first study in India that provides a fair comparison of average costs between public and private providers while taking into account the subsidies public facilities receive from government. The study was able to cover the costs borne by patients as well as the government. The current study suggests a feasible approach for comparing average costs per episode by simultaneously using data from representative surveys of facilities and households.

Further research is recommended to simultaneously compare the cost, quality and health-outcomes associated with different kinds of providers in LMIC contexts. Further research is needed to compare the cost-differentials between providers across health-systems with varying levels of price and provider regulation. Further research is needed to find out why those who utilise private providers make that choice with likelihood of incurring greater OOPE.

Limitations: As reported by earlier studies of Indian hospitals, the current study also faced the challenges involved in collecting detailed data on costs from facilities [6]. Apportioning of total facility cost to a specific function (outpatient care in this case) based on staff time allocation involved approximation though this strategy has been used by many earlier studies. The study was able to control for the type of disease, but the severity of episode could not be measured. The study did not collect data on the procedures or diagnostic-tests used for each patient and they could vary for different providers. Quality of care and medical outcomes are also important aspects for a comparison which the current study could not include in its scope. It has been suggested that greater competition among providers can influence cost [63]. The current study did not examine this aspect.

## Conclusions and recommendations

The current study presents a fair comparison of average cost of care for public providers and the for-profit private providers. The average cost for government and households put together was substantially greater when formal for-profit providers were utilised than the public providers. This has important policy implications for India’s mixed health system, including the desired public-private mix and the appropriate role for for-profit sector. It suggests that purchasing from private providers or extending the publicly funded insurance to outpatient care in India may increase costs. Poor regulation of for-profit providers seems to be an important structural cost driver in mixed health-systems of LMICs like India. Policies to strengthen public provisioning of curative primary care close to communities need to be encouraged to reduce cost.

## Supplementary Information

**Additional file 1.**

**Additional file 2.**

**Additional file 3.**

**Additional file 4.**

**Additional file 5.**

**Additional file 6.**

## Data Availability

The datasets used and/or analyzed during the current study are available from the corresponding author and State Health Resource Centre, Chhattisgarh on reasonable request.

## Abbreviations

CHC: Community Health Centre
CHW: Community Health Worker
DH: District Hospital
INR: Indian Rupee
LMIC: Low and Medium Income Countries
OOPE: Out-of-pocket expenditure
PFHI: Public Funded Health Insurance
PHC: Primary Health Centre
SHC: Sub Health Centre
