# Supplementary material for: Comparing the average cost of outpatient care of public and for-profit private providers in India
Source: BMC Health Serv Res. 2021 Aug 19;21:838. doi: 10.1186/s12913-021-06777-7 (PMC8375109; doi:10.1186/s12913-021-06777-7)
Supplement: Supplementary file 1 — Additional file 1. [file 12913_2021_6777_MOESM1_ESM.docx]

**Additional File S1**

**Note on steps used to compute the average government cost on inputs used by Public facilities per episode of outpatient care**

(The Reference numbers in this note are of references given in the main manuscript)

Input cost data was collected for each type of facility for the year 2019-20.

The main steps in the costing exercise were:

1. All running costs applicable for each kind of government facility were counted.
2. The capital costs were annualised. The value of land of each government facility was taken at current market prices prevalent in the concerned area. The cost of building was taken at current rates of state’s corporation responsible for construction of health facilities. Land was discounted at 7% per annum, considering that the market cost of borrowing capital in India is around 7%. The useful life of buildings was taken as 25 years and building cost was discounted at 11% [18-20]. The useful life of equipments was taken as 5 years and their cost was discounted at 27%.
3. The running costs and the annualized capital costs were added to find the total annual cost.
4. The total annual cost was apportioned for outpatient care based on the average proportion of time spent by facility’s staff on outpatient care activities.
5. The total annual cost on outpatient care of each facility was divided by its total episodes of outpatient care in the year to find the average government cost per episode.

The cost components on which data was collected included - Human Resources, Equipment, Maintenance, Administrative Cost, Medicines, Electricity and Water, Laundry, In-patient Meals, Housekeeping, Ambulance Services, Staff Training, Material and Supplies, Outreach Camps, Travelling Allowances, Stationery, Telephone and Contingencies [25]. The sources of data for each cost centre were facility records, reports and interviews of accounting officers responsible for the facility.

In addition to costs incurred in each facility, the cost of supervision of facility by district authorities was also calculated and apportioned to different types of public facilities. Each district in the state has an office of Chief Medical and Health Officer and a District Programme Management Unit which carries out supervision of public facilities in the district.

Information about human resources working in each type of hospital was collected from hospital duty roster and confirmed from the facility in-charge. Data on their salaries was collected from the concerned accounting officers.

Data on consumption of drugs and consumables was collected from stock registers of store and laboratory and their cost was calculated using rate contract list of the State Medical Services Corporation which carries out bulk purchasing of medical supplies for the state. Cost of reagents, consumables and drugs not supplied through the above Corporation were calculated at prices paid by the district officers responsible for local procurement.

List of equipments and instruments for each type of facility was obtained and their cost was taken at current rates as per corporation rate contracts. Equipments for which such rate contracts were not available, the prices were taken from such rate lists from other states in India. Examples of items in the equipment list included operating table, operating theatre lighting, Boyle’s apparatus, cautery machine, patient examination table, x-ray, ultrasound, ECG machine, C-arm x-ray, defibrillator, ventilator, cardiac monitor, nebuliser, suction apparatus, radiant warmer, pulse oxymeter, diathermy, autoclave, centrifuge, microscope, and water bath. The instruments list includes forceps, scissors, needle holders, and retractors. The furniture list includes beds, almirah (wardrobes), tables, chairs, lockers, and patient trolleys.

Capital expenses were calculated for equipment, ambulances, furniture and fixtures and telephone intercom. The useful life of equipments was taken as five years. Two percent of total cost of equipment was taken as its annual maintenance cost [[19]](https://journals.plos.org/plosone/article?id=10.1371/journal.pone.0069728#pone.0069728-Creese1).

The annual Program Implementation Plan of the state was taken as the base for types of training provided for different cadres and the unit cost of training. Monthly expenditures relating to, Telephone/Internet, Laundry, Housekeeping, Fuel, Water and Electricity and Contingencies etc. were obtained from the accounts office of each type of facility for the reference year (2019-20).

Further, data on outreach or community health work (camps, travel etc.), untied funds, annual maintenance grants were obtained from the office of chief medical and health officer of each district.

The staff belonging to each of the main cadres in public facilities was interviewed to find the average proportion of time spent by them on their three kinds of functions: outpatient care, inpatient care and the non-clinical activities. Based on this, the average proportion of staff time for outpatient care was calculated for each type of public facility [26, 27].

Data on annual number of outpatient episodes were collected from facility records.

Types of Resources used: Data on costs was collected on twenty components. They were classified into six main types of resources. The cost according to the type of resource used per episode of outpatient care is given in the table below:

Table: **Cost per episode by type of resource in public facilities**

| **Facility** | **Cost (in INR) per episode by type of resource in public facilities** | | | | | | |
| --- | --- | --- | --- | --- | --- | --- | --- |
|  | **Human Resources** | **Medicines** | **Diagnostics** | **Infrastructure** | **Utilities** | **Others** | **Total** |
| DH | 173 | 53 | 10 | 46 | 19 | 5 | 305 |
| CHC | 179 | 45 | 9 | 101 | 33 | 4 | 373 |
| PHC | 181 | 42 | 8 | 93 | 24 | 8 | 358 |
| SHC | 72 | 15 | 3 | 77 | 7 | 10 | 184 |
| CHW | 44 | 2 | 1 | 0 | 0 | 9 | 55 |
| **All Public Facilities** | **105 (53%)** | **21 (11%)** | **5 (3%)** | **48 (24%)** | **12 (6%)** | **8 (4%)** | **198** |

**Average Government Cost on Inputs used by Public facilities per episode of Outpatient Care provided (2019-20):**

1. **Community Health Worker (CHW):**

**Table: Average Government Cost per episode of outpatient care by CHWs (n=50 CHWs)**

| Cost Components | Average Cost (INR) | % of total cost |
| --- | --- | --- |
| Human Resource (Remuneration Payments to CHWs) | 43800 | 77% |
| Training Cost | 2100 | 4% |
| Drugs and Rapid tests | 2142 | 4% |
| Supervision Cost | 9000 | 16% |
| **Total** | **57042** | **100%** |
| Share of Outpatient Care in Time Use of CHWs | | 15% |
| Annual Episodes of Outpatient Care | | 158 |
| Government Cost per episode of Outpatient Care (INR) | | 54 |

**2. Sub Health Centre (SHC):**

**Table: Government Cost per episode of outpatient care by SHCs**

**(n=25 SHCs)**

| Cost Components | Cost (INR) | % of total cost |
| --- | --- | --- |
| Human Resource | 421200 | 39.74 |
| Annualized cost of land | 103219 | 9.74 |
| Annualized building Cost | 348750 | 32.91 |
| Drugs and diagnostics | 112046 | 10.57 |
| Contingencies | 24000 | 2.26 |
| Stationary and furniture annual cost | 18000 | 0.17 |
| Cost of supervision by district authorities | 48787 | 4.6 |
| **Total** | **1076003** | **100** |
| Share of Outpatient Care in Time Use of SHC Staff | | 16.3% |
| Annual Episodes of Outpatient Care | | 954 |
| Government Cost per episode of Outpatient Care (INR) | | 184 |

**3. Primary Health Centre (PHC):**

**Table: Government Cost per episode of outpatient care by PHCs**

**(n=20 PHCs)**

| Cost Components | Cost (INR) | % of total cost |
| --- | --- | --- |
| Human Resource | 2880600 | 48.22% |
| Annualized building Cost | 848572 | 14.21% |
| Annualized cost of land | 257785 | 4.32% |
| Medicines | 677875 | 11.35% |
| Furniture, Fixture and others | 419338 | 7.02% |
| Materials, consumables & supplies | 204600 | 3.43% |
| Staff Training | 147020 | 2.46% |
| Electricity & Water | 108000 | 1.81% |
| Outreach camps | 84000 | 1.41% |
| Laundry | 41566 | 0.70% |
| Contingencies | 40000 | 0.67% |
| Building Maintenance | 40000 | 0.67% |
| Telephone (Operational) | 24000 | 0.40% |
| House keeping | 20492 | 0.34% |
| Equipment Maintenance | 17540 | 0.29% |
| Traveling Allowances | 15000 | 0.25% |
| Stationery | 12000 | 0.20% |
| Telephone/ Intercom | 3000 | 0.05% |
| Cost of supervision by district authorities | 132200 | 2.21% |
| Total cost | 5973588 | 100 |
| Share of Outpatient Care in Time Use of PHC Staff | | 46.5% |
| Annual Episodes of Outpatient Care | | 7765 |
| Government Cost per episode of Outpatient Care (INR) | | 358 |

**4. Community Health Centre (CHC):**

**Table: Government Cost per episode of outpatient care by CHCs**

**(n=15 CHCs)**

| Cost Components | Cost (INR) | % of total cost |
| --- | --- | --- |
| Human Resource | 15907008 | 44.49% |
| Annualized building Cost | 5864000 | 16.40% |
| Annualized cost of land | 1700400 | 4.76% |
| Medicines | 4067248 | 11.38% |
| Furniture, Fixture and others | 1316026 | 3.68% |
| Ambulance(Operational Cost) | 1516800 | 4.24% |
| Materials & Supplies | 1227600 | 3.43% |
| Staff Training | 1170120 | 3.27% |
| Outreach camps | 609600 | 1.70% |
| Electricity & Water | 720000 | 2.01% |
| Laundry | 249396 | 0.70% |
| Contingencies | 240000 | 0.67% |
| Building Maintenance | 240000 | 0.67% |
| Telephone (Operational) | 144000 | 0.40% |
| House keeping | 122952 | 0.34% |
| Equipment Maintenance | 105242 | 0.29% |
| Traveling Allowances | 90000 | 0.25% |
| Stationery | 72000 | 0.20% |
| Telephone/ Intercom | 19200 | 0.05% |
| Cost of supervision by district authorities | 374400 | 1.05% |
| Total cost | 35755992 | 100.00% |
| Share of Outpatient Care in Time Use of CHC Staff | | 37.5% |
| Annual Episodes of Outpatient Care | | 35950 |
| Government Cost per episode of Outpatient Care (INR) | | 373 |

**5. District Hospital (DH):**

**Table: Government Cost per episode of outpatient care by DHs**

**(n=5 DHs)**

| Cost Components | Cost (INR) | % of total cost |
| --- | --- | --- |
| Human Resource | 49930560 | 54.02% |
| Annualized building Cost | 1922400 | 2.08% |
| Annualized cost of land | 7080600 | 7.66% |
| Medicines | 13557494 | 14.67% |
| Materials, Consumables & Supplies | 5913600 | 6.40% |
| Ambulance(Operational Cost) | 3866400 | 4.18% |
| Staff Training | 2247720 | 2.43% |
| Electricity & Water | 1800000 | 1.95% |
| Furniture, Fixture and others | 900000 | 0.97% |
| Outreach camps | 744000 | 0.80% |
| Equipment Maintenance | 421027 | 0.46% |
| Contingencies | 600000 | 0.65% |
| Building Maintenance | 600000 | 0.65% |
| House keeping | 456592 | 0.49% |
| Laundry | 429619 | 0.46% |
| Stationery | 240000 | 0.26% |
| Telephone (Operational) | 172800 | 0.19% |
| Traveling Allowances | 90000 | 0.10% |
| Telephone/ Intercom | 36000 | 0.04% |
| Cost of supervision by district authorities | 1420800 | 1.54% |
| Total cost | 92429612 | 100% |
| Share of Outpatient Care in Time Use of DH Staff | | 41.4% |
| Annual Episodes of Outpatient Care | | 125310 |
| Government Cost per episode of Outpatient Care (INR) | | 305 |
