# Supplementary material for: Comparing the average cost of outpatient care of public and for-profit private providers in India
Source: BMC Health Serv Res. 2021 Aug 19;21:838. doi: 10.1186/s12913-021-06777-7 (PMC8375109; doi:10.1186/s12913-021-06777-7)
Supplement: Supplementary file 2 — Additional file 2. [file 12913_2021_6777_MOESM2_ESM.docx]

**Additional File S2:**

**Table – Socio-economic and Demographic Profile of Individuals covered in Household Survey**

| **Characteristic** | **Category** | **N=8286** |
| --- | --- | --- |
| Place of sample (Urban/Rural) | Urban | 1708 (20.6%) |
|  | Rural | 6578 (79.4%) |
| Household size | Mean with 95% CI | 5.96 (5.91-6.10) |
|  | Median | 6 |
| Occupation | Government Job | 459 (5.5%) |
|  | Private Job with Fixed Monthly Salary | 433 (5.2%) |
|  | Self-employed in agriculture | 3749 (45.3%) |
|  | Self-employed in non-agriculture | 570 (6.8%) |
|  | Informal worker | 888 (10.7%) |
|  | Unemployed | 2176 (26.3%) |
| Caste | ST | 2769 (33.4%) |
|  | SC | 984 (11.9%) |
|  | OBC | 4202 (50.7%) |
|  | Others | 276 (3.3%) |
| Per-capita monthly Household Consumption Expenditure in Quintiles (Range in INR) | Q5- Richest (>15001) | 1616 (19.5%) |
|  | Q4- Rich (1001 to 15000) | 1045 (12.6%) |
|  | Q3-Middle (834 to 1000) | 936 (11.6%) |
|  | Q2- Poor (601 to 833) | 2766 (33.45) |
|  | Q1- Poorest (Up to 600) | 1864 (22.5%) |
| Sex of Individual | Male | 4071 (49.1%) |
|  | Female | 4214 (50.9%) |
| Age Category of Individual | 0-1 Years | 61 (0.7%) |
|  | 1-4 Years | 616 (7.4%) |
|  | 5-14 Years | 1677 (20.2%) |
|  | 15-29 Years | 2456 (29.6%) |
|  | 30-44 Years | 1606 (19.4%) |
|  | 45-59 Years | 1118 (13.5%) |
|  | 60+ Years | 711 (8.6%) |
| Education of Individual | No Literate | 2449 (29.6%) |
|  | Pre-Primary | 1014 (12.2%) |
|  | Primary | 1205 (14.5%) |
|  | High school | 2379 (28.8%) |
|  | Graduation and above | 1217 (14.7%) |
| Individuals whose HH has health insurance | PMJY | 5206 (62.8%) |
|  | MSBY | 2098 (25.3%) |
|  | Other | 73 (0.9%) |
|  | No Insurance | 896 (10.8%) |
| Individuals enrolled under any Insurance |  | 5649 (68.2%) |
| Household Head | Male | 7034 (84.9%) |
|  | Female | 1252 (15.1%) |
| Age of Household Head | Mean with 95% CI | 47.4 (47.2-47.7) |
|  | Median | 47 |
|  | Age Up to 60 | 6999 (84.5%) |
|  | Above 60 | 1279 (15.5%) |
| Religion | Hindu | 8001 (96.6%) |
|  | Muslim | 140 (1.7%) |
|  | Christian | 127 (1.5%) |
|  | Sikh | 13 (0.2%) |
| Type of House | Katcha | 4643 (56.3%) |
|  | Semi-pakka | 1570 (19%) |
|  | Pakka | 2039 (24.7%) |
| Nearest Government Health Facility | Mean distance (in Km) with 95% CI | 6.3 (6.1- 6.4) |
|  | Median | 4 Km |
| Drinking Water Facility | Tap inside House | 3019 (36.5%) |
|  | Outside House /Community tap or well | 1232 (14.9%) |
|  | Hand pump | 3364 (40.7%) |
|  | Well | 625 (7.6%) |
|  | Others | 27 (0.3%) |
| Toilet Facility | Toilet in Own House | 7228 (87.4%) |
|  | Free Community Toilet | 189 (2.3%) |
|  | Paid Community Toilet | 40 (0.5%) |
|  | Open Field/Drain/ Road Etc | 783 (9.5%) |
|  | Others | 27 (0.3%) |
| Primary Source of energy for Cooking | Do not Cook | 78 (0.9%) |
|  | Wooden Logs | 5661 (68.3%) |
|  | Gas | 2199 (26.5%) |
|  | Cow Dung | 42 (0.5%) |
|  | Coal | 153 (1.8%) |
|  | Electricity | 4 (0.05%) |
|  | Others | 41 (0.5%) |
