# Supplementary material for: Comparing the average cost of outpatient care of public and for-profit private providers in India
Source: BMC Health Serv Res. 2021 Aug 19;21:838. doi: 10.1186/s12913-021-06777-7 (PMC8375109; doi:10.1186/s12913-021-06777-7)
Supplement: Supplementary file 3 — Additional file 3. [file 12913_2021_6777_MOESM3_ESM.docx]

**Supplementary File S3**

**Sensitivity Analysis: Government cost for episode of outpatient care in public facilities**

Sensitivity Analysis was carried out for government cost in public facilities. For this purpose, the assumptions were changed to create two scenarios different from the base scenario. One scenario was with decrease the variable by 30%. The other scenario involved increased the variable by 30% from the base level.

The government cost under the three scenarios is given in the table below:

**Table:** **Government cost for episode of outpatient care in public facilities in different scenarios**

| **Variable** | **Government cost (in INR) for episode of outpatient care in public facilities in different scenarios** | | |
| --- | --- | --- | --- |
|  | By decreasing the variable by 30% | Base Level | By increasing the variable by 30% |
| Discount rate for Land | 195 | 198 | 201 |
| Discount rate for Building | 187 | 198 | 209 |
| Discount rate for equipments | 196 | 198 | 200 |
| **Proportion of Human Resources cost apportioned to outpatient care** | **167** | **198** | **229** |
| Proportion of Medicines cost apportioned to outpatient care | 192 | 198 | 204 |
| **Proportion of Infrastructure cost apportioned to outpatient care** | **184** | **198** | **212** |

The above table shows that compared to other variables, a change in Human Resources and Infrastructure produced greater change in the cost than the other variables.

Apart from the above scenarios, if the government spending on diagnostics were to be increased fivefold (from the actual level of 3% to 15%), it will increase the government cost per episode to INR 233.
