# Supplementary material for: Comparing the average cost of outpatient care of public and for-profit private providers in India
Source: BMC Health Serv Res. 2021 Aug 19;21:838. doi: 10.1186/s12913-021-06777-7 (PMC8375109; doi:10.1186/s12913-021-06777-7)
Supplement: Supplementary file 4 — Additional file 4. [file 12913_2021_6777_MOESM4_ESM.docx]

**Supplementary File S4**

**Process Map of Outpatient Care in Public Facilities (in Chhattisgarh)**

Patient Leaves

**Patient Registration**

**Laboratory**

Diagnostics tests

Patient enters

**Consultation Room**

Consultation by Physican/Nurse

Examination by Nurse

Diagnostic

Prescribed

(Yes/No)

**Pharmacy**
