# Supplementary material for: Comparing the average cost of outpatient care of public and for-profit private providers in India
Source: BMC Health Serv Res. 2021 Aug 19;21:838. doi: 10.1186/s12913-021-06777-7 (PMC8375109; doi:10.1186/s12913-021-06777-7)
Supplement: Supplementary file 5 — Additional file 5. [file 12913_2021_6777_MOESM5_ESM.docx]

**Supplementary File S5**

**Disease category wise Mean OOPE for different types of providers**

| **Disease category wise Mean OOPE (INR) with 95% CI for different types of providers** | | | | |
| --- | --- | --- | --- | --- |
| **Type of Disease** | **Overall** | **Public providers** | **Formal For-Profit Providers** | **Informal Private Providers** |
| Communicable Diseases | 829 (609-1050) | 192 (99-285) | 2094 (1413-2775) | 487 (379-595) |
| NCDs | 1656 (401-2913) | 144 (31-258) | 1783 (886-2680) | 352 (234-469) |
| Maternal Care | 675 (0-1449) | 147 (0-354) | 845 (315-1374) | 600 (100-1300) |
| Injuries | 1872 (0-5096) | 190 (0-429) | 3520 (40-7430) | 563 (312-813) |
| Others | 2856 (425-5288) | 377 (0-761) | 332 (55-609) | 282 (115-448) |
