# Supplementary material for: Comparing the average cost of outpatient care of public and for-profit private providers in India
Source: BMC Health Serv Res. 2021 Aug 19;21:838. doi: 10.1186/s12913-021-06777-7 (PMC8375109; doi:10.1186/s12913-021-06777-7)
Supplement: Supplementary file 6 — Additional file 6. [file 12913_2021_6777_MOESM6_ESM.docx]

**Additional File S6**

**Linear Regression for size of OOPE**

Number of obs = 796

R-squared = 0.22

| **OOPE** | **Coef.** | **Std. Err.** | **p value** | **[95% Conf. Interval]** | |
| --- | --- | --- | --- | --- | --- |
| **Type of Provider** |  |  |  |  |  |
| Public Provider |  |  |  |  |  |
| Formal Private Provider | 2034.17 | 266.55 | 0.00 | 1510.91 | 2557.44 |
| Informal Private Provider | 227.99 | 224.11 | 0.31 | -211.96 | 667.94 |
| **Household Size** | 34.46 | 45.02 | 0.44 | -53.93 | 122.85 |
| **Social Group (Caste)** |  |  |  |  |  |
| ST | 1 |  |  |  |  |
| SC | -47.03 | 349.32 | 0.89 | -732.78 | 638.72 |
| OBC | 55.78 | 241.72 | 0.82 | -418.74 | 530.30 |
| Others | 1610.91 | 638.09 | 0.01 | 358.26 | 2863.56 |
| **Sex** |  |  |  |  |  |
| Male | 1 |  |  |  |  |
| Female | -19.66 | 189.77 | 0.92 | -392.20 | 352.88 |
| **Place** |  |  |  |  |  |
| Urban | 1 |  |  |  |  |
| Rural | 291.53 | 286.06 | 0.31 | -270.02 | 853.09 |
| **Occupation** |  |  |  |  |  |
| Government Job | 1 |  |  |  |  |
| Private Job with Fixed Monthly Salary | 142.13 | 629.56 | 0.82 | -1093.77 | 1378.03 |
| Self employed in agriculture | 17.80 | 453.61 | 0.97 | -872.68 | 908.28 |
| Self employed in non agriculture | 416.21 | 571.62 | 0.47 | -705.95 | 1538.37 |
| Informal worker | 157.28 | 530.59 | 0.77 | -884.33 | 1198.89 |
| Unemployment | 33.55 | 477.73 | 0.94 | -904.29 | 971.39 |
| **Education** |  |  |  |  |  |
| Not literate | 1 |  |  |  |  |
| Below Primary | 416.74 | 332.64 | 0.21 | -236.27 | 1069.76 |
| Primary | 864.47 | 324.90 | 0.01 | 226.66 | 1502.28 |
| High School | 710.57 | 315.59 | 0.03 | 91.03 | 1330.12 |
| 12th/ Diploma and Above | 469.28 | 415.27 | 0.26 | -345.93 | 1284.50 |
| **AGE** |  |  |  |  |  |
| 1-4 Years | 1 |  |  |  |  |
| 5-14 Years | -786.67 | 338.46 | 0.02 | -1451.09 | -122.24 |
| 15-29 Years | -1033.12 | 399.94 | 0.01 | -1818.25 | -247.98 |
| 30-44 Years | -477.25 | 380.32 | 0.21 | -1223.87 | 269.36 |
| 45-59 Years | -572.38 | 380.12 | 0.13 | -1318.59 | 173.84 |
| 60 and Above | 107.07 | 406.17 | 0.79 | -690.29 | 904.42 |
| **Per Capita Household Exp. Quintile** |  |  |  |  |  |
| Poorest | 1 |  |  |  |  |
| Poor | -153.60 | 294.75 | 0.60 | -732.22 | 425.03 |
| Middle | 27.33 | 296.54 | 0.93 | -554.81 | 609.47 |
| Rich | -167.03 | 288.47 | 0.56 | -733.33 | 399.26 |
| Richest | -363.90 | 292.97 | 0.22 | -939.03 | 211.23 |
| **Disease** |  |  |  |  |  |
| Cold and Cough | 1 |  |  |  |  |
| Malaria | -175.78 | 278.17 | 0.53 | -721.87 | 370.30 |
| Diarrhoea | -382.17 | 473.99 | 0.42 | -1312.67 | 548.33 |
| Body pain | 296.01 | 484.48 | 0.54 | -655.08 | 1247.10 |
| Skin Infection | -37.37 | 535.09 | 0.94 | -1087.81 | 1013.07 |
| Typhoid | 2763.34 | 561.05 | 0.00 | 1661.93 | 3864.75 |
| Knee and Joint pain | -434.68 | 997.05 | 0.66 | -2392.00 | 1522.64 |
| Weakness | 1760.38 | 876.52 | 0.05 | 39.66 | 3481.09 |
| Injuries | 2049.40 | 983.59 | 0.04 | 118.51 | 3980.29 |
| Respiratory Infection | 1582.00 | 1167.85 | 0.18 | -710.63 | 3874.63 |
| Stomach ache | 5185.68 | 883.31 | 0.00 | 3451.65 | 6919.71 |
| Others | 2027 | 839 | 0.02 | 381 | 3673 |
| **House type** |  |  |  |  |  |
| Kutcha | 1.00 |  |  |  |  |
| Semi-Pakka | -13.50 | 267.04 | 0.96 | -537.73 | 510.73 |
| Pakka | -99.95 | 265.61 | 0.71 | -621.37 | 421.48 |
| _cons | -47.13 | 658.07 | 0.94 | -1339.00 | 1244.74 |
